# Ultrafine Particle Distribution and Chemical Composition Assessment during Military Operative Trainings

**DOI:** 10.3390/ijerph14060579

**Published:** 2017-05-30

**Authors:** Marcello Campagna, Ilaria Pilia, Gabriele Marcias, Andrea Frattolillo, Sergio Pili, Manuele Bernabei, Ernesto d’Aloja, Pierluigi Cocco, Giorgio Buonanno

**Affiliations:** 1 Department of Medical Sciences and Public Health, University of Cagliari, 09042 Monserrato, Italy; mam.campagna@gmail.com (M.C.); gabriele.marcias@libero.it (G.M.); serginho.pili@gmail.com (S.P.); ernestodaloja@gmail.com (E.d.A.); pcocco@unica.it (P.C.); 2 Department of Civil and Environmental Engineering and Architecture, University of Cagliari, via Marengo 2, 09123 Cagliari, Italy; andrea.frattolillo@unica.it; 3 Chemistry Department, Test Flight Centre, IAF, Pratica di Mare AFB, 00040 Pomezia, Italy; manuele.bernabei@aeronautica.difesa.it; 4 Department of Civil and Mechanical Engineering, University of Cassino and Southern Lazio, I-03043 Cassino, Italy; buonanno@unicas.it; 5 International Laboratory for Air Quality and Health, Queensland University of Technology (QUT), 4001 Brisbane, Australia; 6 Department of Engineering, University of Naples “Parthenope”, 80133 Naples, Italy

**Keywords:** ultrafine particles, environmental exposure, monitoring, electric low-pressure impactor (ELPI+), military training, emissions

## Abstract

(1) Background: The assessment of airborne particulate matter (PM) and ultrafine particles (UFPs) in battlefield scenarios is a topic of particular concern; (2) Methods: Size distribution, concentration, and chemical composition of UFPs during operative military training activities (target drone launches, ammunition blasting, and inert bomb impact) were investigated using an electric low-pressure impactor (ELPI+) and a scanning electron microscope (SEM), equipped with energy-dispersive spectroscopy (EDS); (3) Results: The median of UFPs, measured for all sampling periods and at variable distance from sources, was between 1.02 × 10^3^ and 3.75 × 10^3^ particles/cm^3^ for drone launches, between 3.32 × 10^3^ and 15.4 × 10^3^ particles/cm^3^ for the ammunition blasting and from 7.9 × 10^3^ to 1.3 × 10^4^ particles/cm^3^ for inert launches. Maximum peak concentrations, during emitting sources starting, were 75.5 × 10^6^ and 17.9 × 10^6^ particles/cm^3^, respectively. Particles from the drone launches were predominantly composed of silicon (Si), iron (Fe) and calcium (Ca), and those from the blasting campaigns by magnesium (Mg), sulphur (S), aluminum (Al), iron (Fe), barium (Ba) and silicon (Si); (4) Conclusions: The investigated sources produced UFPs with median values lower than other anthropogenic sources, and with a similar chemical composition.

## 1. Introduction

Particulate matter (PM) is defined as “a disperse system of liquid or solid small particles suspended in a gas” [1]. The ultrafine particle (UFP) fraction includes particles with diameter less than 100 nm, generated by photochemical processes and combustion, or originating from various natural and anthropogenic sources, which are widely present in the living and working environments [2,3].

UFPs from anthropogenic activities, such as combustion phenomena, industrial processes and occupational activities, urban traffic and heating-related emissions, and indoor sources contribute the most to the airborne concentration in the general environment [4].

In the last years, several epidemiological studies have linked exposure to airborne particulates to adverse health effects, and particularly cardiovascular and respiratory diseases. The International Agency for Research on Cancer (IARC) recently classified particulate matter, a major component of outdoor air pollution, as a human carcinogen (Group 1) [5]. In particular, the ability of UFPs to easily cross the biological barriers and their greater biological activity were recognized as their main characteristics leading to harmful health consequences [6,7,8,9,10,11]. Therefore, several studies have recently been conducted to quantify and characterize UFP emissions from different sources and their distribution in the environment [12,13,14,15,16,17].

The assessment of occupational exposure to particulate matter (PM) in the battlefield scenarios is a topic of particular concern, as soldiers may be exposed to particles generated by multiple sources, such as ballistic impacts, blasting, motor vehicles and aircraft operations [18]. 

The majority of the studies conducted on battlefield or military scenarios have focused on measurement of microparticles through gravimetric techniques followed by chemical characterization [19], while only few studies investigated airborne UFP emissions during selected military activities [20,21,22]. Such studies were performed with several UFP detection techniques, for example using a condensation particle counter (CPC), fast mobility particle sizer (FMPS) and aerodynamic particle sizer spectrometer (APS). However, to the best of our knowledge, only a few studies [23] used an electric low-pressure impactor (ELPI), which is considered especially useful for characterizing UFP emissions in occupational settings due to its ability to make real time measurements of particle size distribution and to collect particle for morphological and chemical analysis [13,24,25,26].

Aim of our study was to assess UFP emissions during the military training activities conducted in the Quirra Interforce Firing Range (QIFR), which is located in southeastern Sardinia, and provides technical and logistical support for training to North Atlantic Treaty Organization (NATO) military forces. The main military activities realized in this location were described by Cristaldi et al. (2013) [27].

Several studies have addressed the environmental pollution following the QIFR military activities, and the potential health effects on the nearby general population. Radioactive pollutants, electromagnetic fields and toxic elements or compounds in various matrices have been investigated [28,29], including metals and other trace elements in biological and inorganic matrices [27]. In addition, gravimetric particulate PM_10_ and PM_2.5_ monitoring during rocket test and blasting operations in the same areas were conducted [28]. However, UFP emissions during military experimental activities have not been investigated thus far.

## 2. Materials and Methods 

### 2.1. Trials

**Figure 1:**
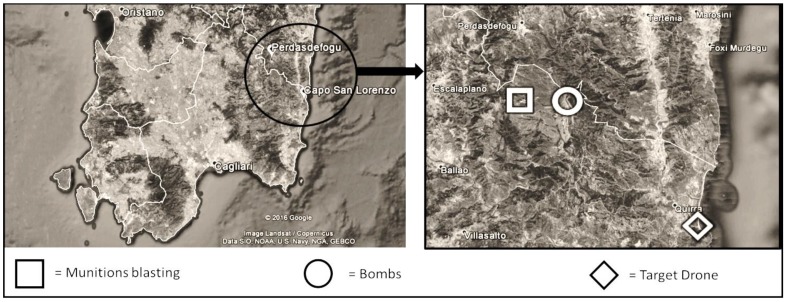
Geographical map of the Quirra Interforce Firing Range (QIFR); on the right, the approximate location of the military activities is described.

The QIFR is composed of two different areas, a seaside range (Capo San Lorenzo, Villaputzu) and an inland range (Perdasdefogu). Figure 1 illustrates the QIFR’s geographic location, and the approximate location of the sampling sites.

#### 2.1.1. Drone Take Off

On 16 April 2012, 21 February 2013, and 11 February 2015, stationary measurements were conducted in the seaside range, during three radio-controlled target drone take-off, at a horizontal distance of 42, 7 and 20 m from the take-off area, respectively.

A target drone is an aerial vehicle, in this case radio-controlled from the ground, used to test anti-aircraft systems. The drone is composed of a main engine and two lateral boosters for the propulsion. Launch operations lasted around 5 min from engine ignition to take off, while measurements were prolonged to at least 90 min (maximum 4 h). 

The stationary sampler position was chosen to be downwind to the drone ramp whenever possible, but for the third sampling the position was upwind (wind direction: South-Southwest, SSW, wind maximum speed <18.5 km/h), and closer to the target drone ramp. Electricity for the stationary sampler was supplied by an outlet in the control shelter to comply with operational safety requirements. 

#### 2.1.2. Ammunition Blasting

During spring 2015, six stationary measurements were conducted in the inland range, during an obsolete ammunition (tracer, armor-piercing and incendiary bullets) blasting campaign on 17 April, 25 May, and 26 May. 

The detonation area was composed of a reinforced concrete box, in which some sandbags were placed surrounding the obsolete ammunitions and the plastic explosive.

The ammunition blasting activity was conducted in five phases: ammunition and explosive preparation in the detonation area; a safety area check (500 m around the detonation box); detonation; and, finally, following a thirty-minute wait, a second safety area check; and area watering with a tanker truck for ten minutes for dust breakdown. 

The sampling duration during such operation was at least 1 h. Table 1 describes the sites of the stationary sampler. 

Stationary sampling sites were selected to be downwind at increasing distance from the source (from 10 to 400 m), depending on the operational needs and to preserve the instruments’ security.

Electricity for the stationary sampler was supplied by a Pramac ES 8000 three-phase power unit, equipped with Honda GX 390 petrol engine, located at least 50 m downwind from the sampler.

#### 2.1.3. LIZARD-Guided Inert Bomb Launch

On 26 and 27 May 2016, stationary samplings were conducted in the ground range during two LIZARD-guided inert air-ground launches, at a horizontal distance of 100 and 40 m from the target, respectively. 

LIZARD is a laser target system, used during bomb launch. The training with the LIZARD guide consists of an airplane launch of an inert bomb that simulates the mechanical and electronic parts of a bomb. 

A few seconds after launch, the inert bomb collided with a designed target in the ground. The impact is followed by a safety area check and area watering with a diesel tanker truck for dust breakdown. 

The stationary sampler position was selected to ensure the safety of instruments and to be downwind from the target whenever possible. Electricity was supplied by a Pramac E4000 power unit, equipped with petrol engine, located at least 70 m downwind from the sampler. The sampling duration during this operation was at least 4 h. Table 2 describes the sites of the stationary sampler, meteorological conditions. 

### 2.2. Analysis

An ELPI+™ (Dekati, Tampere, Finland) device was used to count the number and size, surface area, and mass of the particulate matter. Description of the ELPI+ function and its principles of operation have been reported [30,31,32]. Briefly, the ELPI+ makes real-time measurements of the particles in the 6 nm–10 μm size range, and it collects them on substrates in 14 size range fractions [33]. A vacuum pump is used to control the airflow through the instrument (0.6 m^3^/h, pressure of 40 mbar). 

The substrate composition was aluminum foil during the target drone launch and on 17 April for the first blasting sampling, and polycarbonate for the rest of blasting campaign and during inert bomb launch samplings. 

UFP count was calculated as the sum of particles collected by the ELPI+ first four stages (6–94 nm). The assumed density value was 1g/cm^3^, due to the different composition of airborne PM and the gap of knowledge about the real density of collected aerosols. This parameter has a relatively weak influence on the count because it affects only the size range interval for each impactor stage [13]. 

During each sampling, any ground activity, such as motor vehicle passages, was noted by a trained operator.

Data analysis were conducted through the Microsoft Excel spreadsheet provided by Dekati Ltd.

After blasting, inert bomb and target drone launch operations are characterized by very rapid changes in particles size and concentration; the parameters were measured in 10-s time resolution.

Information about wind direction and speed was derived from the meteorological station of the firing range, and it was used for the experimental design and data analysis. 

A qualitative analysis of the UFPs collected during drone launches and ammunition blasting was conducted by SEM (SUPRA™ 35 with GEMINI column technology, Carl Zeiss, Oberkochen, Germany) with energy-dispersive spectroscopy (EDS; NCA, Oxford Instruments, Abingdon, UK). It was not possible to conduct this analysis on samples collected during inert bomb launches, due to technical reasons.

## 3. Results

### 3.1. Particle Count

#### 3.1.1. Drone Take-Off

**Figure 2:**
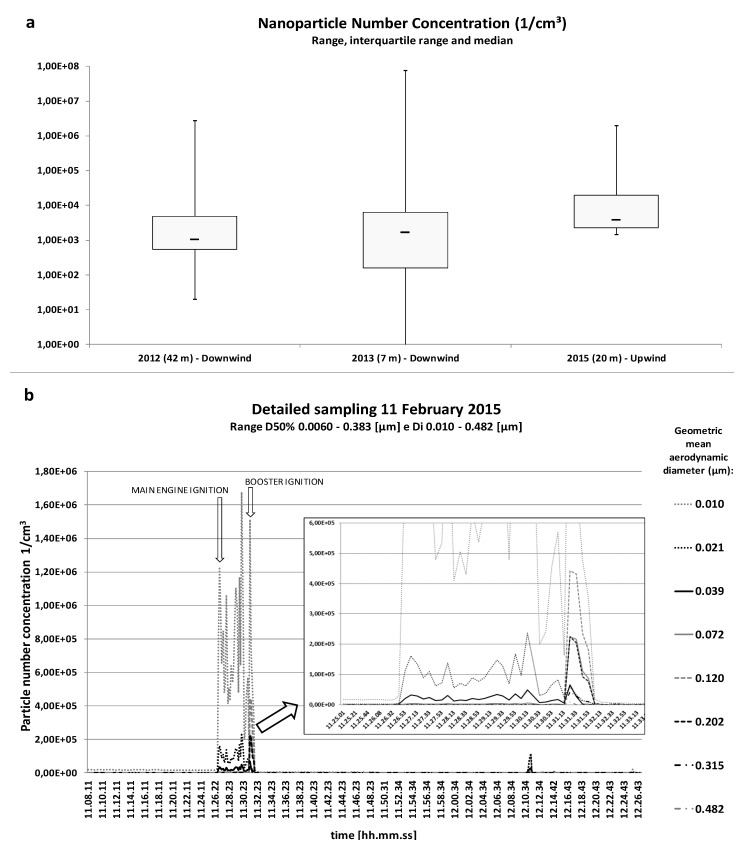
Particle count during the target drone launch (**a**). The lower picture shows (**b**) a representative time plot. Aerodynamic diameter range = D50%. Geometric mean aerodynamic diameter range = Di.

Figure 2 shows UFP count during target drone take-off. The median UFP concentrations in 2012, 2013 and 2015 samples were 1.0 × 10^3^, 1.6 × 10^3^, and 3.8 × 10^3^ particles/cm^3^, respectively, versus 7.9 × 10^2^, 3.4 × 10^3^, and 2.0 × 10^4^ particles/cm^3^, respectively, before the launch. Peak values in UFP concentration in all samplings ranged from 2.0 × 10^6^ to 7.6 × 10^7^ particles/cm^3^. Both median values and peak values decreased with the increasing distance of the sampling site from the source. Arithmetic mean values were respectively 1.52 × 10^4^, 4.0 × 10^5^ and 6.0 × 10^4^ particles/cm^3^, and geometric mean values were 1.48 × 10^3^, 1.19 × 10^3^ and 7.21 × 10^3^ particles/cm^3^.

The lower picture in Figure 2 shows the particle count by time since target drone launch during the sampling on 11 February 2015. The UFP generation process during the target drone launch is instantaneous. After the first peak, during the main engine ignition, UFP concentration ranged from 1.9 × 10^5^ to 2.0 × 10^6^ particles/cm^3^ for almost 6 min. A weaker peak in the final stage of the launch, for larger particles with geometric mean aerodynamic diameter between 120 and 315 nm, was probably caused by the booster ignition or coagulation and condensation processes. After the operations, with the exception of two peaks generated by motor vehicles approaching, dilution of UFPs prevailed in coagulation and condensation processes since no shift in larger particle count in was detected.

#### 3.1.2. Ammunition Blasting

**Figure 3:**
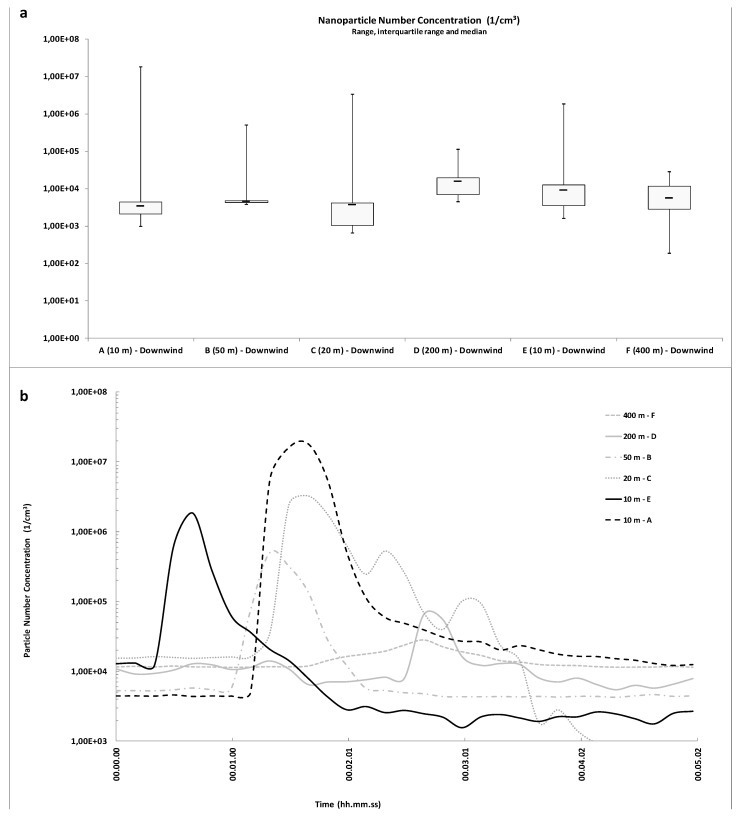
Ultrafine particle (UFP) count at the six sampling sites during inert bomb launch: (**a**) statistics represented through box plots; (**b**) total particle count versus time.

The pattern of particle count by location with respect to the source and time since emission in the other circumstances is alike. Figure 3 shows UFP count during the ammunition blasting campaign. The median UFP count at the A, B, C, D, E and F sites were 3.3 × 10^3^, 4.5 × 10^3^, 3.6 × 10^3^, 1.5 × 10^4^, 8.9 × 10^3^ and 5.5 × 10^3^ particles/cm^3^, respectively. Median values before the blasting were 4.2 × 10^3^, 4.6 × 10^3^, 1.6 × 10^4^, 1.4 × 10^4^, 1.3 × 10^4^ and 1.0 × 10^4^ particles/cm^3^, respectively. Arithmetic mean values were 1.33 × 10^5^, 6.79 × 10^3^, 1.90 × 10^4^, 1.43 × 10^4^, 1.40 × 10^4^ and 7.19 × 10^3^ particles/cm^3^, respectively, and geometric mean values were 3.84 × 10^3^, 4.74 × 10^3^, 3.45 × 10^3^, 1.23 × 10^4^, 7.12 × 10^3^ and 5.47 × 10^3^ particles/cm^3^, respectively.

The peak UFP concentration in all samplings ranged from 2.8 × 10^4^ to 1.8 × 10^7^ particles/cm^3^. Higher peaks were observed in the samples from the “A, C and E”, 10–50 m downwind from the detonation box (Figure 3b), after less than 2 min from the blast. By increasing the distance between the ammunition blasting site and the sampling instrument (Samplings D and F), we observed a relevant decrease of the peaks as well an increase of the time up to about 3 min after blasting (Figure 3b). After the blast, the UFP count returned to the initial level. Several lower peaks were observed at the end of each sampling period due to motor vehicles passage.

#### 3.1.3. LIZARD-Guided Inert Bomb Launch

**Figure 4:**
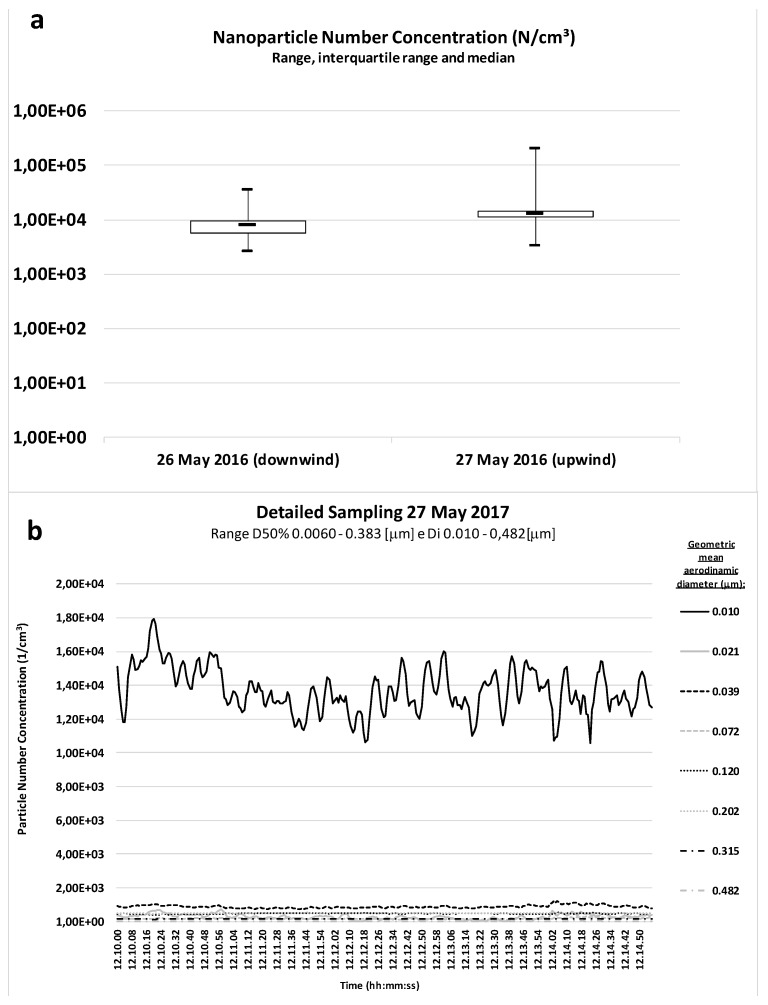
Particles number during the inert bomb launch (**a**). The lower picture (**b**) shows a representative UFPs count distribution by time of the day.

Figure 4 shows UFP count during the inert bomb launches. The median UFP concentrations in the samplings on 26 and 27 May were 7.9 × 10^3^ and 1.3 × 10^4^ particles/cm^3^, respectively. Arithmetic mean values were 7.80 × 10^3^ and 1.33 × 10^4^ particles/cm^3^, and geometric mean values were 7.42 × 10^3^ and 1.28 × 10^4^ particles/cm^3^, respectively.

Peak UFP concentration in all samples ranged from 3.6 × 10^4^ to 2.1 × 10^5^ particles/cm^3^. Weak peak readings were detected before and after launch, due to the passage of a diesel tanker truck for area watering and a power unit ignition during this operation. No substantial fluctuation in particle count, except for a one-minute slight increase in particles of the 21 nm and 39 nm diameter, were observed at the ground inert collision (minute 12.12.01), as observed in the lower picture in Figure 4.

### 3.2. Size Distribution

**Figure 5:**
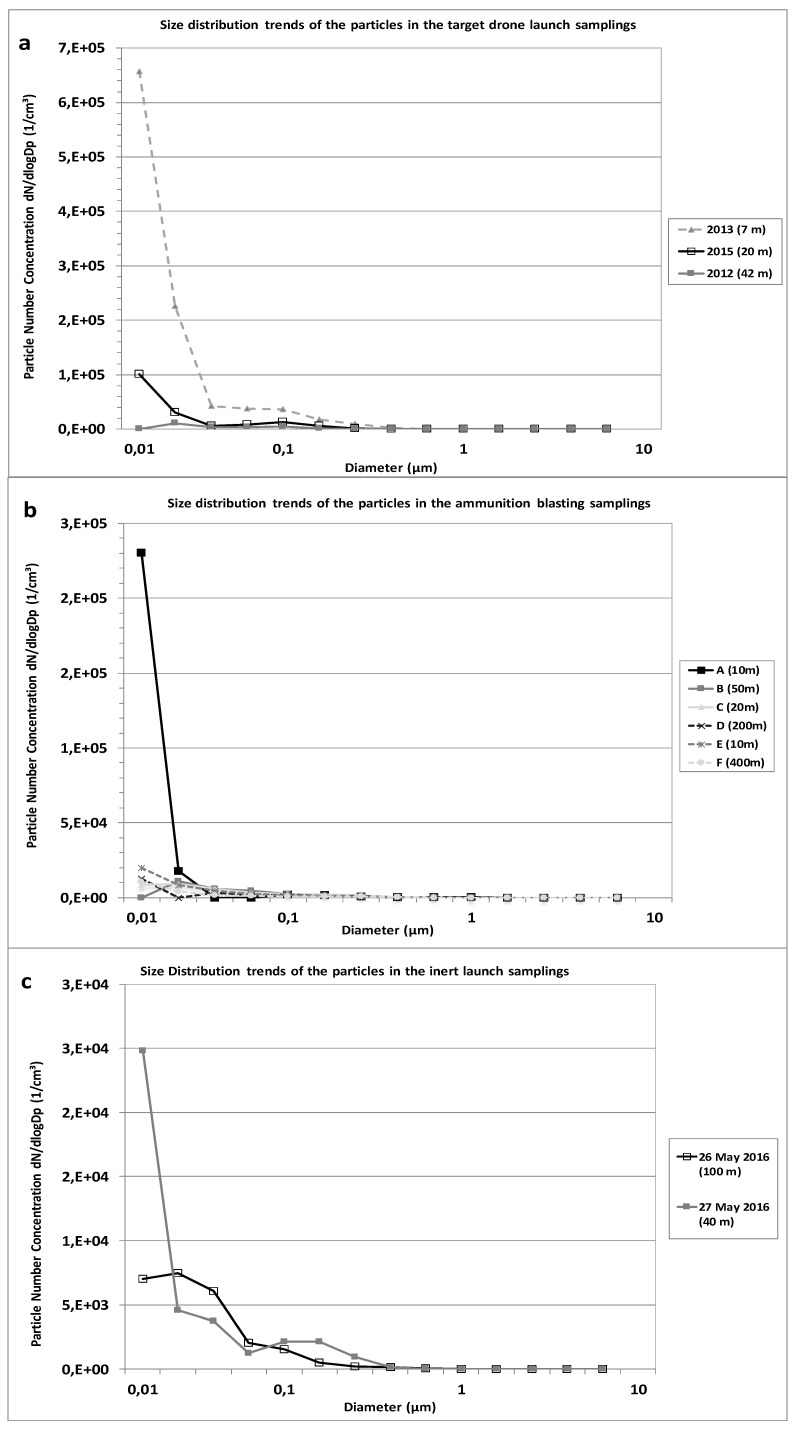
Size distribution patterns during the target drone launches (**a**); ammunition blasting campaign (**b**) and inert launches (**c**).

Figure 5 shows the size distribution of the particulate matter for inert bomb and target drone take-off, as well as for ammunition blasting. During the target drone take-off, we observed considerable emissions of UFPs (93% of the total particles number concentration were in the size range of 10–72 nm), consistent with the main engine ignition, followed by the two lateral booster ignition after several minutes. However, with a different distribution pattern, ammunition blasting also produced UFPs predominantly in the size range between 10 and 39 nm. A similar pattern was observed during the inert bomb air-ground launch.

### 3.3. SEM-EDS Analysis

**Figure 6:**
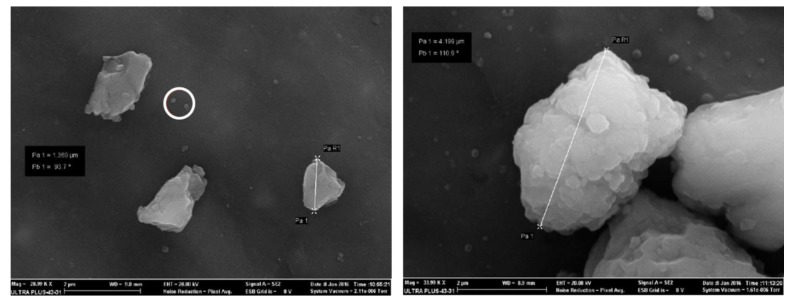
SEM analysis of the particles collected on 11 February 2015, during a drone launch exercise.

The morphology of deposited particles observed by SEM varied from single spherical primary particles (Figure 6, white circle) to irregular aggregates. Aggregates observed by SEM in filters had different size ranges, independently of the ELPI+ stage of collection. This could be related to the formation of particle aggregates or to the accumulation of an excess of particles in the filters after the sampling. 

EDS elemental analysis showed that particles collected by ELPI+ during the drone launch were predominantly composed by silicon (Si), iron (Fe) and calcium (Ca). Particles collected during the ammunition blasting campaign were mainly composed of magnesium (Mg), sulfur (S), aluminum (Al), iron (Fe), barium (Ba) and, to a lower extent, silicon (Si). 

**Figure 7:**
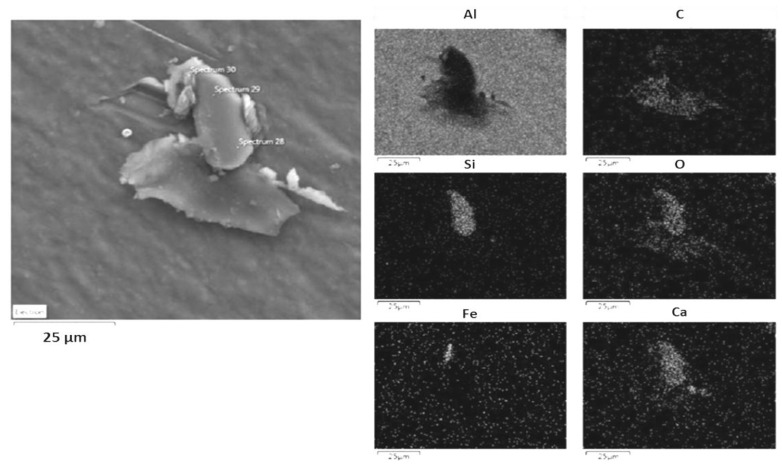
SEM analysis of the particles aggregates collected on 11 February 2015, 20 m from the drone launch site. The chemical mapping by energy-dispersive spectroscopy (EDS) of major elements (Al, C, Si, O, Fe, and Ca) is shown in the right pictures.

**Figure 8:**
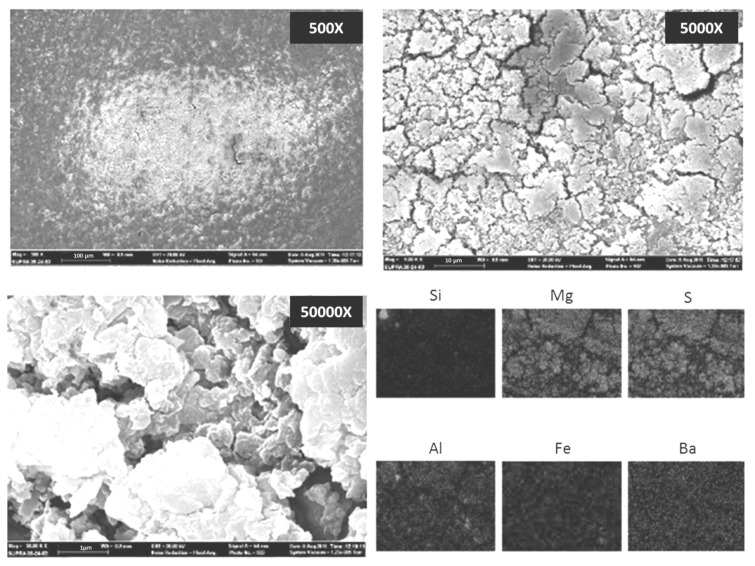
SEM analysis of the particle aggregates collected during ammunition blasting in the “C” sampling site, 20 m from the detonation box. Their chemical composition mapping EDS is showed in the bottom-right pictures.

Figure 7 and Figure 8 show two representative examples of the morphology of aggregates collected respectively during a target drone launch and ammunition blasting, and their chemical mapping. Aluminum shown in the composition mapping in the Figure 7 seems to be derived from the substrate used for this sampling.

## 4. Discussion

In our study, UFP counts immediately after the main engine ignition and during all drone launch operations were similar to that observed during pre-flight aircraft operations in an adjacent position nearby the airstrip of a military aviation base [21]. The emission from the drone engines included mainly particles with aerodynamic diameter ranging from 10 to 72 nm, comparable to those in the proximity of military aircraft engines (25 nm) and measured in a general aviation airport (size mode about 11 nm) [15,21]. Our findings confirm the inverse relation between particle count and distance from the source observed in other studies [15,34]. 

The median particle count during target drone take off, inert bomb launches and ammunition blasting was in the same order of magnitude as that measured downwind from the jet engine emissions in a military aviation base, and one order of magnitude lower of the background values in a typical exposure of urban pedestrians [21,23,35,36]. In addition, geometric mean values measured during all samplings were well lower than those measured by a fast mobility particle sizer (FMPS) during Polyvinyl Chloride (PVC ) welding and concrete work in a tunnel rehabilitation work, and similar to exposure of the main surgeon during some electrosurgery activities [37,38].

Time change and size distributions of UFP count following ammunition-blasting operations were similar to those observed during high-speed impact of inert bombs; some differences in the peak readings were related to the variable distances of the stationary sampler from the detonation box [20,22].

During target drone launches and ammunition blasting, the median UFP count over the whole sampling period was not different from that before either the launch or the blast; on the other hand, during inert bomb launches peaks in UFPs count were observed in relation with impact to the ground. This would suggest that peak UFP counts had a poor influence in median concentrations, mainly in relation to the very short duration of the events.

Several studies showed a relation between wind conditions and UFP count [15,21]. We could not confirm such a relation, due to the extreme difference in sampling conditions, and, in some instances, the need to comply with operational and security requirements lead to the decision on where to locate the sampling device.

We expected differences in PM size distribution in the three different activities. In particular, we expected a larger fraction of small size particles during target drone launches and ammunition blasting, because of higher temperatures and energy-release processes (completely fuel combustion and explosion). During ammunition blasting, we did not expect non-combustion processes, such as high velocity impacts of the fragments generated by the blast against the structure of the detonation box (sand bags), to contribute in small size particle generation, because such fragments are retained within the box protection structure. Instead, in inert bomb launches, we expected generation of particles in the large size range, because of the mechanical impact of the inert to the ground from high altitude. However, previous studies suggested a potential UFP production during high-speed mechanical impacts [22].

We did not carry out background UFP measurements, due to the logistic requirements (such as transport and energy), and their consistency with operational needs and territorial features. However, given the temporal characteristics of the events (instantaneous and with rapid resolution), the characterization of the pattern of UFP emission and decay is only partially affected by the lack of specific background measurements. 

We can draw some conclusion from our measurements during ammunition blasting:

1. The median values are slightly influenced by the ammunition blasting campaign because of the effect of the atmospheric dilution that lowers the particle count quite rapidly;
2. The peak UFP counts occur immediately after the ammunition blast, within 50 m downwind from it, and they last for a short time [39];
3. At a distance of 200–400 m the effect of the blast in terms of peak UFP count seems negligible, not only in respect to the background median values, but also in terms of absolute short-term exposure. In fact, the peak UFP counts we detected at such distance from the emission sources were lower than the concentrations typically measured in urban areas [40], and well lower than those observed in indoor microenvironments [41].

The EDS elemental analysis of particles collected during the three types of activities showed a chemical composition similar to that generated by other anthropogenic and natural sources [21,42]. 

Engelbrecht et al. (2009) [19] showed that trace metals (arsenic, lead, antimony and zinc) were concentrated in the PM_2.5_ fraction, while geological components (aluminum, manganese, calcium and magnesium) prevailed in larger fractions. Our study did not confirm these findings, since the same portion of geological components (such as iron, silicon, calcium and aluminum aggregates) were identified in all observed samples, independent on the size fraction.

The expected chemical composition of samples collected during target drone launches consisted predominantly in particles produced by marine aerosol and fuel combustion residues. During the ammunition blasting, we expected to detect explosives, obsolete ammunition residuals, and traces of sand used as protection in the detonation box. Unfortunately, we did not receive information about the chemical composition nor the type of fuel of the target drone, nor the explosives and ammunitions used in these operational activities. Besides, we were unable to conduct soil analysis. Without such information, it was not possible to make inference about particle origin.

Analysis conducted by “*Agenzia regionale per la protezione dell'ambiente della Sardegna*” (ARPAS) in the seaside range of the QIFR territory in 2012 during rocket tests showed increased concentrations of arsenic, aluminum, barium, phosphorus, sodium and potassium in airborne total particles; in the inland range, during blasting operations, increased values of arsenic, nickel and chromium five times the national limits were found [28]. In our study, we have not conducted a quantitative analysis of airborne particles, but qualitative determination performed by SEM showed a particle composition different to that observed by ARPAS in both the seaside and inland range.

## 5. Conclusions

To the best of our knowledge, this is the first study that uses an ELPI+ impactor device to investigate UFP emissions during military activities. The ELPI+ is a resistant instrument, able to operate over a particle size ranging three order of magnitude, to take real-time measurements and to collect samples in substrates for chemical analysis. In particular, it allows for data about particle number and distribution to be collected, as well as information on morphological and chemical properties. This could aid a better comprehension of the extent to which military experimental activities contribute to airborne pollutants emissions and possible related adverse health effects.

The sources we investigated produced a substantial amount of UFPs, similar to that measured in a military aviation base [21], but with median values over the sampling period lower than anthropogenic sources such as urban traffic, and with a similar chemical composition.

The major part of our samplings was located near the training activities, in areas where the military personnel would not operate during operations due to safety requirements. Furthermore, only one stationary sampling instrument was used, so that we could not assess occupational exposure at safe distances from the emitting source. Further studies should assess personal exposure in order to better-characterize health risks following occupational exposure among the military, and environmental exposure among the resident population living nearby the military firing ranges.

## Figures and Tables

**Table 1:** Sampling time, sampler position and meteorological conditions during the ammunition blasting campaign.

ID	Day	Start (Hour)	End (Hour)	Distance, Position from the Source	Wind Direction—Max Speed (km/h)	Blasting Time (Hour)
A	17 April	9:32	10:31	10 m, E (downwind *)	W 44.5	9:39
B	17 April	11:07	12:27	50 m, E (downwind *)	W 44.5	11:39
C	25 May	8:58	11:09	20 m, W (downwind *)	SE 29.6	9:16 and 10:02
D	25 May	14:45	16:32	200 m, W (downwind *)	SE 29.6	14:47 and 16:04
E	26 May	8:52	11:00	10 m, E (downwind *)	W 46.3	9:24 and 10:02
F	26 May	14:08	16:17	400 m, E (downwind *)	W 46.3	14:40 and 15:49

* Electric low-pressure impactor (ELPI+) position related to wind direction. Wind directions: East (E), West (W), South-East (SE).

**Table 2:** Sampling time, sampler position and meteorological conditions during inert bomb launches.

ID	Day	Start (Hour)	End (Hour)	Distance, Position from the Source	Wind Direction—Max Speed (km/h)	Launch Time (Hour)
I	26 May	11:21	15:21	100 m, WNW (downwind *)	SE 29.6	14:50:11
II	27 May	09:24	17:42	40 m, WNW (upwind *)	W 24.1	12:11:52

* ELPI+ position related to wind direction. Wind directions: West-Northwest (WNW)

## References

[1] Vincent J.H. (2007). Part A: Scientific framework for aerosol sampling. Aerosol Sampling: Science, Standards, Instrumentation and Applications.

[2] Marconi A. (2006). Fine, ultrafine and nano-particles in the living and working setting: Potential health effects and measurement of inhalation exposure. G. Ital. Med. Lav. Ergon..

[3] Vincent J.H., Clement C.F. (2000). Ultrafine particles in workplace atmospheres. Philos. Trans. Math. Phys. Eng. Sci..

[4] Morawska L., Keogh D.U., Thomas S.B., Mengersen K.L. (2008). Modality in ambient particle size distributions and its potential as a basis for developing air quality regulation. Atmos. Environ..

[5] Straif K., Cohen A., Samet J. (2013). Air Pollution and Cancer.

[6] Kreyling W.G., Semmler-Behnke M., Möller W. (2006). Health implications of nanoparticles. J. Nanopart. Res..

[7] Pope C.A., Dockery D.W. (2006). Health effects of fine particulate air pollution: Lines that connect. J. Air Waste Manag. Assoc..

[8] Pietroiusti A. (2012). Health implications of engineered nanomaterials. Nanoscale.

[9] Pedata P., Garzillo E.M., Sannolo N. (2010). Ultrafine particles and effects on the body: Review of the literature. G. Ital. Med. Lav. Ergon..

[10] Cesaroni G., Badaloni C., Gariazzo C., Stafoggia M., Sozzi R., Davoli M., Forastiere F. (2013). Long-term exposure to urban air pollution and mortality in a cohort of more than a million adults in Rome. Environ. Health Perspect..

[11] Buonanno G., Marks G.B., Morawska L. (2013). Health effects of daily airborne particle dose in children: Direct association between personal dose and respiratory health effects. Environ. Pollut..

[12] Ott W.R., Wallace L.A., McAteer J.M., Hildemann L.M. (2017). Fine and ultrafine particle exposures on 73 trips by car to 65 non-smoking restaurants in the San Francisco Bay Area. Indoor Air.

[13] Kero I., Naess M.K., Tranell G. (2015). Particle Size Distributions of Particulate Emissions from the Ferroalloy Industry Evaluated by Electrical Low Pressure Impactor (ELPI). J. Occup. Environ. Hyg..

[14] Mazaheri M., Clifford S., Jayaratne R., Megat Mokhtar M.A., Fuoco F., Buonanno G., Morawska L. (2014). School children’s personal exposure to ultrafine particles in the urban environment. Environ. Sci. Technol..

[15] Hu S., Fruin S., Kozawa K., Mara S., Winer A.M., Paulson S.E. (2009). Aircraft emission impacts in a neighborhood adjacent to a general aviation airport in Southern California. Environ. Sci. Technol..

[16] Stabile L., Scungio M., Buonanno G., Arpino F., Ficco G. (2016). Airborne particle emission of a commercial 3D printer: The effect of filament material and printing temperature. Indoor Air.

[17] Scungio M., Vitanza T., Stabile L., Buonanno G., Morawska L. (2017). Characterization of particle emission from laser printers. Sci. Total Environ..

[18] Falvo M.J., Osinubi O.Y., Sotolongo A.M., Helmer D.A. (2015). Airborne hazards exposure and respiratory health of Iraq and Afghanistan veterans. Epidemiol. Rev..

[19] Engelbrecht J.P., McDonald E.V., Gillies J.A., Jayanty R.K., Casuccio G., Gertler A.W. (2009). Characterizing mineral dusts and other aerosols from the Middle East—Part 2: Grab samples and re-suspensions. Inhal. Toxicol..

[20] Stabile L., Iannitti G., Vigo P., Ruggiero A., Russi A., Buonanno G. (2014). Ultrafine particle generation by high-velocity impact of metal projectiles. J. Phys. Conf. Ser..

[21] Buonanno G., Bernabei M., Avino P., Stabile L. (2012). Occupational exposure to airborne particles and other pollutants in an aviation base. Environ. Pollut..

[22] Buonanno G., Stabile L., Ruggiero A., Iannitti G., Bonora N. Ultrafine particle size distribution during high velocity impact of high density metals. Proceedings of the AIP Conference.

[23] Campagna M., Frattolillo A., Pili S., Marcias G., Angius N., Mastino C.C., Cocco P., Buonanno G. (2016). Environmental Exposure to Ultrafine Particles inside and nearby a Military Airport. Atmosphere.

[24] Kero I.T., Jørgensen R.B. (2016). Comparison of Three Real-Time Measurement Methods for Airborne Ultrafine Particles in the Silicon Alloy Industry. Int. J. Environ. Res. Public Health.

[25] Klejnowski K., Krasa A., Rogula-Kozłowska W., Błaszczak B. (2013). Number size distribution of ambient particles in a typical urban site: The first Polish assessment based on long-term (9 months) measurements. Sci. World J..

[26] Meléndez A., García E., Carnicer P., Pena E., Larrión M., Legarreta J.A., Gutiérrez-Cañas C. (2010). Fine and ultrafine emission dynamics from a ferrous foundry cupola furnace. J. Air Waste Manag. Assoc..

[27] Cristaldi M., Foschi C., Szpunar G., Brini C., Marinelli F., Triolo L. (2013). Toxic Emissions from a Military Test Site in the Territory of Sardinia, Italy. Int. J. Environ. Res. Public Health.

[28] Agenzia Regionale per la Protezione Ambientale Della Sardegna (ARPAS), Programma di Monitoraggio Ambientale del Poligono Interforze del Salto di Quirra (PISQ) (2012). Stato di Avanzamento Delle Attività Dell’ARPAS Nella Supervisione del Programma di Monitoraggio Ambientale del PISQ.

[29] Istituto Zooprofilattico Sperimentale Della Sardegna (IZS) (2011). Relazione Sui Risultati Preliminari dei Piani di Monitoraggio Sugli Inquinanti Ambientali Nell’area del Poligono Interforze del Salto di Quirra. http://www.izs-sardegna.it/doc_news/08-11-2011_Relazione_conferenza_stampa.pdf.

[30] Marjamäki M., Keskinen J., Chen D.R., Pui D.Y.H. (2000). Performance evaluation of the electrical low-pressure impactor (ELPI). J. Aerosol Sci..

[31] Marjamäki M., Lemmetty M., Keskinen J. (2005). ELPI response and data reduction I: Response functions. Aerosol Sci. Technol..

[32] Lemmetty M., Keskinen J., Marjamäki M. (2005). The ELPI response and data reduction II: Properties of kernels and data inversion. Aerosol Sci. Technol..

[33] (2012). ELPI+TM User Manual—Version 1.2.

[34] Hsu H.H., Adamkiewicz G., Houseman E.A., Vallarino J., Melly S.J., Wayson R.L., Spengler J.D., Levy J.I. (2012). The relationship between aviation activities and ultrafine particulate matter concentrations near a mid-sized airport. Atmos. Environ..

[35] Buonanno G., Fuoco F., Stabile L. (2011). Influence parameters on particle exposure of pedestrians in urban microenvironments. Atmos. Environ..

[36] Kumar P., Morawska L., Birmili W., Paasonen P., Hu M., Kulmala M., Harrison R.M., Norford L., Britter R. (2014). Ultrafine particles in cities. Environ. Int..

[37] Jørgensen R.B., Buhagen M., Føreland S. (2016). Personal exposure to ultrafine particles from PVC welding and concrete work during tunnel rehabilitation. Occup. Environ. Med..

[38] Ragde S.F., Jørgensen R.B., Føreland S. (2016). Characterisation of Exposure to Ultrafine Particles from Surgical Smoke by Use of a Fast Mobility Particle Sizer. Ann. Occup. Hyg..

[39] Manigrasso M., Stabile L., Avino P., Buonanno G. (2013). Influence of measurement frequency on the evaluation of short-term dose of sub-micrometric particles during indoor and outdoor generation events. Atmos. Environ..

[40] Scungio M., Arpino F., Stabile L., Buonanno G. (2013). Numerical simulation of ultrafine particle dispersion in urban street canyons with the Spalart-Allmaras turbulence model. Aerosol Air Qual. Res..

[41] Buonanno G., Johnson G., Morawska L., Stabile L. (2011). Volatility characterization of cooking-generated aerosol particles. Aerosol Sci. Technol..

[42] Bzdek B.R., Horan A.J., Pennington M.R., Janechek N.J., Baek J., Stanier C.O., Johnston M.V. (2014). Silicon is a frequent component of atmospheric nanoparticles. Environ. Sci. Technol..

